# Supplementary material for: HENNOVATION: Learnings from Promoting Practice-Led Multi-Actor Innovation Networks to Address Complex Animal Welfare Challenges within the Laying Hen Industry
Source: Animals (Basel). 2019 Jan 11;9(1):24. doi: 10.3390/ani9010024 (PMC6356511; doi:10.3390/ani9010024)
Supplement: Supplementary file 1 [file animals-09-00024-s001.pdf]

Table S1. Level of engagement / activity within each of the 19 networks as perceived by the network's facilitator during the final workshop (May 2017).

		Not completed	Low	Moderate	High
**General indicators relevant to the function of each network**					
0.1	Level of participant enthusiasm and energy of the network	0	1	4	14
0.2	Level of participant trust and knowledge sharing within the network	0	1	4	14
0.3	Level of facilitators intervention (rate of intervention facilitator)	0	7	8	4
0.4	Level or frequency of interaction with support actors (engagement of the relevant actor at the right time)	0	5	6	8
Overall general engagement		**0 (0%)**	**14 (18%)**	**22 (29%)**	**40 (53%)**
**Step 1 Problem identification**					
1.1	Level of clarity of purpose and shared objective as a network	0	0	4	15
1.2	Level of agreement on network function (e.g. decision making, common rules, reaching consensus etc.)	0	2	4	13
1.2	Degree to which the problem identified was based on shared need (common problem)	0	1	5	13
1.3	Market or other actors value the problem (relevance)	0	0	3	16
1.4	Capacity of network to find practical solutions to the problem identified (perceived capacity of the network by the facilitator)	0	1	6	12
Overall engagement in step 1		**0 (0%)**	**4 (4%)**	**22 (23%)**	**69 (73%)**
**Step 2 Generation of ideas**					
2.1	Level of which the idea/solution is shared by the network	0	1	3	15
2.2	Feasibility of the idea (includes financially viable, based on ADAS tool)	10	0	4	5
2.3	Level of diversity of knowledge (resources) used: science, advisor's input, practical experience etc.	0	2	3	14
2.4	Capacity of network to trial the practical solutions selected (perceived capacity of the network by the facilitator	**0**	**1**	**4**	**14**
Overall engagement in step 2		***10 (13%)***	***4 (5%)***	***14 (18%)***	***48 (63%)***
**Step 3 Action planning & resource mobilization**					
3.1	Robustness of innovation action planning including time-frame and task division (everyone knows what is happening, when and by whom)	2	4	4	9
3.2	Level of clarity on anticipated result (research question) and system or criteria in place for to measure and monitor the results (e.g. viability)	1	1	9	8
3.3	Level of resources the members within the network commit towards trialling.	1	4	7	7
3.4	Level of external support (whether scientific, from industry or technical)	1	4	5	9
Overall engagement in step 3		***5 (7%)***	***13 (17%)***	***25 (33%)***	***33 (43%)***
**Step 4 Practical trialing and development**					
4.1	Level and rate of innovation - action plan leads to action.	5	1	4	9

4.2	Willingness to discuss and share within the network successes and failures (to learn from failures)	4	2	3	10
Overall engagement in step 4		***9 (24%)***	***3 (8%)***	***7 (18%)***	***19 (50%)***
**Step 5 Implementation and up-scaling**					
5.1	Level of satisfaction of members with regard to relevance and affordability of solutions developed.	9	0	1	9
5.2	Number of network members applying the innovation as common practice across their farm.	14	0	2	3
5.3	Network members' pride of what they achieved (wanting to share and scale -up the innovative idea).	9	1	0	9
Overall engagement in step 5		***32 (56%)***	***1 (2%)***	***3 (5%)***	***21 (37%)***
**Step 6 Dissemination**					
6.1	Network has actively sought to disseminate innovation beyond network members	12	2	1	4
6.2	Innovation has been subsequently adopted by other actors and bodies	19	0	0	0
Overall engagement in step 6		***31 (82%)***	***2 (5%)***	***1 (3%)***	***4 (11%)***
